# Influence of hand-arm self-avatar motion delay on the directional perception induced by an illusory sensation of being twisted

**DOI:** 10.1038/s41598-022-10543-y

**Published:** 2022-04-22

**Authors:** Tomohiro Amemiya

**Affiliations:** 1 Virtual Reality Educational Research Center, The University of Tokyo, Tokyo, 1138656 Japan; 2 Graduate School of Information Science and Technology, The University of Tokyo, Tokyo, 1138656 Japan

**Keywords:** Perception, Human behaviour

## Abstract

Sensory information from movements of body parts can alter their position when exposed to external physical stimuli. Visual information monitors the position and movement of body parts from an exterior perspective, whereas somatosensory information monitors them from an internal viewpoint. However, how such sensory data are integrated is unclear. In this study, a virtual reality (VR) system was used to evaluate the influence of the temporal difference between visual and somatosensory information from hand movements on the directional perception of a torque while modifying the visual appearance (human hand vs. non-human object) and visuohaptic congruency (congruent vs. incongruent) of self-avatars. Visual information was provided by the movement of the self-avatars in a VR environment, while somatosensory information was provided by vibrations with asymmetrical amplitudes that gave the participants the sensation of being continuously pushed or pulled without actually moving any body part. Delaying the movement of the avatar by 50 ms resulted in the sensitivity of the force direction perception to be lower with human hands than with non-human avatars, whereas a delay of 200 ms resulted in a higher sensitivity. This study can contribute to applications requiring multisensory integration in a VR environment.

## Introduction

In virtual reality (VR) systems, the self-avatar is viewed from the first- or third-person perspective via a head-mounted display (HMD), providing synchronized visuomotor feedback to the user. A method that synchronizes body motions provides an immersive experience by intuitively allowing users to move their hands and arms. Congruent visual and somatosensory feedback creates a sense of embodiment, which refers to the feeling of being inside, controlling, and having a virtual body^1,2^. The sense of embodiment can be decomposed into three components: the sense of self-location, the sense of agency, and the sense of ownership. Among them, the senses of body ownership and agency are the two that most fundamentally impact how we consciously experience our bodies: the former refers to feeling an artificial body or body part as one’s own, and the latter to the experience of initiating and controlling their actions and consequences^3–6^.

Moreover, the appearance of a self-avatar and whether it is similar to the human body, influences not only the sense of embodiment^7–9^, especially the ownership component^10–12^, but also the performance of the user in locomotion and object interaction tasks^13^, thus, implying that the appearance of a self-avatar plays an important role in integrating the stimuli presented to a self-body.

In addition to the appearance of the self-avatar, the temporal mismatch between its movements and those of its natural counterpart undermines the senses of body ownership^14^ and agency^15^, and the delay between visual and somatosensory feedback affects the self-body recognition^16–18^. In a study by Di Luca et al., the temporal mismatch between the movements of a natural hand and those of its corresponding self-avatar when touching an object was noticed when haptic feedback was presented more than 50 ms after visual feedback^19^. On the contrary, Maselli et al. reported that the temporal window for visuotactile integration of body related cues expanded when illusory ownership was induced^20^.

Regarding spatial mismatch between self-avatar and natural hand movements, visuomotor congruence between real and virtual body movements highly contribute to strengthen the sense of agency^21^, whereas discrepancies between visual and motor information tend to diminish it^22^. In addition to the visuomotor congruence, the congruence in visual-proprioceptive information significantly improves the haptic discrimination of force directions^23^. These intersensory conflicts can be explained by Bayes-optimal cue integration^24^. For instance, visual and tactile information is optimally integrated to improve discrimination of the velocity of self-hand movements^25^. Since visual sensation takes a perceptual preference over other senses in many contexts, vision is dominant when multiple stimuli have the same perceived intensity^26^, or when a conflicting stimulus with twice the intensity of the visual data is presented^27^. Users in a VR environment are more sensitive to spatial mismatches between visual positions of the self-avatar and those of the natural hand than to visual-proprioceptive discrepancies between them^28^.

Taken together, the backgrounds presented above indicate that it is still unclear how the appearance of the self-avatar and the spatial and temporal mismatch between self-avatar and natural hand movements affect the integration of visual and somatosensory information. The present study aimed to elucidate how the delay between the self-avatar motion and force feedback affects the perception of the force direction. Within-subject experiments were conducted, where participants viewed their hand-arm avatars in a VR environment, while using different combinations of anthropomorphism (human-like vs. non-human-like), visuohaptic congruency (congruent vs. incongruent), and visuohaptic delays. In this work, it was hypothesized that the participants who used human-like hand avatars were more likely affected by a visuohaptic delay. By creating the sensation of being twisted through the application of asymmetric oscillations instead of an actual force, the sensitivity of the force direction perception while observing the hand-arm self-avatar was analyzed.

## Results

An experiment was performed to investigate the effects of the delay between visual and somatosensory stimuli and the congruency between self-avatar motion and force feedback on the judgment of the force direction perceived. The participants wearing an HMD and holding a haptic device were asked to judge the twisting direction perceived while watching the movements of self-avatars. Moreover, the effect of the visual appearance of the self-avatars on the perception of the rotational direction was examined by displaying two types of avatars, a human hand or a branch, in the VR environment. Signal detection theory was employed to quantify the perceptual sensitivity of motion direction (i.e., the information loss on motion direction), and analyzed subjective scores for body ownership and sense of agency using a questionnaire.

### Sensitivity of direction discrimination

**Figure 1:**
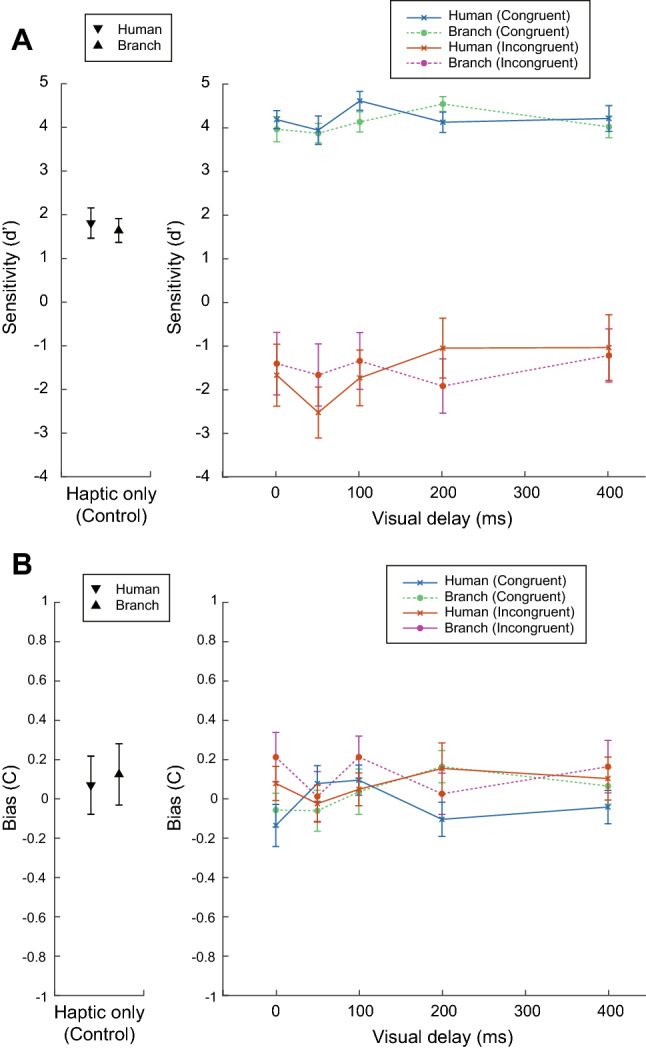
Mean (**A**) sensitivity (d′) and (**B**) response bias (C) of direction discrimination.

Figure 1A,B show the mean sensitivity of direction discrimination and mean post-perceptual response bias (i.e., tendency to perceive all stimuli as moving ‘clockwise’ or ‘counter clockwise’ irrespective of the actual direction), respectively. Without visual movement (i.e., in the haptic-only control condition), the participants’ mean sensitivity for the human and branch avatars was 2.46 and 1.69, respectively, because the haptic stimuli amplitude threshold for each participant was adjusted to ensure a reliability between 70 and 80%. The sensitivity dropped when incongruent movements (opposite the visual and haptic stimuli) were presented and increased with congruent movements for every level of delay.

A three-way repeated-measure ANOVA was conducted on the aligned ranks for the sensitivity (d′) considering the within-subject factors of hand avatar type (human or non-human), congruency (congruent and incongruent vision-haptic stimuli), and delay (0, 50, 100, 200, or 400 ms). The main effects of congruency (*F*(1,26) = 96.76, *p* < 0.001, $\eta _{p}^{2}$ = 0.79) and delay (*F*(4,104) = 3.82, *p* = 0.006, $\eta _{p}^{2}$ = 0.13) were statistically significant, unlike that of hand avatar type (*F*(1,26) = 0.21, *p* = 0.65, $\eta _{p}^{2}$ = 0.008). More importantly, the three-way interaction was significant (*F*(4,104) = 4.03, *p* = 0.004, $\eta _{p}^{2}$ = 0.13); but not so the other possible interactions (*p* > 0.20). A simple interaction test was performed to analyze the three-way interaction. The results showed that the avatar type × delay under an incongruent condition was significant (*F*(4,104) = 2.77, *p* = 0.031, $\eta _{p}^{2}$ = 0.096). Then, a simple-simple main effect test was conducted for the significant simple interaction. For the avatar type × delay under an incongruent condition, the simple-simple main effect of the avatar type was significant for a delay of 50 ms (*F*(1,26) = 5.64, *p* = 0.025, $\eta _{p}^{2}$ = 0.18). On the contrary, the simple-simple main effect of the delay was significant for the human hand avatar (*F*(4,104) = 3.05, *p* = 0.020, $\eta _{p}^{2}$ = 0.11).

The results from post hoc pairwise comparisons showed that the mean for the 50 ms delay was significantly smaller than that for 200 ms when presenting a human hand avatar and incongruent conditions (*t*(104) = 3.76, adjusted *p* = 0.003).

Similarly, a three-way repeated-measures ANOVA was conducted on the aligned ranks for the response bias (criterion). The main effect of congruency (*F*(1,26) = 6.20, *p* = 0.02, $\eta _{p}^{2}$ = 0.19) was statistically significant, but no significant differences were found in the other main effects or interactions.

### Subjective questionnaires

**Figure 2:**
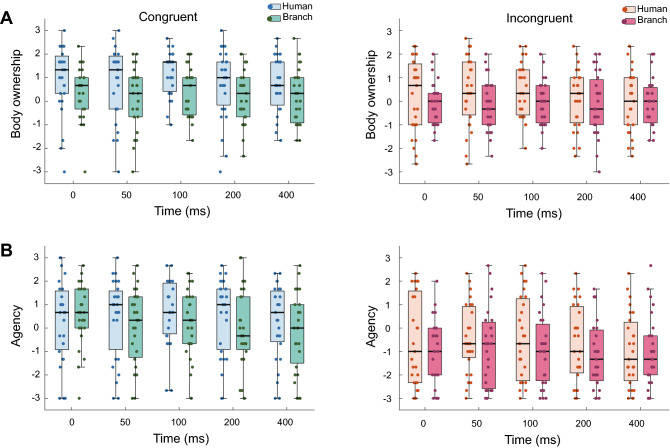
Boxplots for body ownership (**A**) and sense of agency (**B**).

Figure 2 shows the synthesized subjective ratings obtained from the questionnaire for the senses of body ownership and agency. After inverting the answers to the reverse scale questions, the answers for each item were aggregated and averaged to compute the score for each experimental block per participant. Participants experienced a body ownership in the congruent condition using a human avatar, with a maximum median score of 1.5. The body ownership statements were positively rated through all congruent conditions (medians exceeded zero), but not through all incongruent conditions (medians were less than + 0.5). Participants experienced a sense of agency in the congruent condition with a human avatar (maximum median scores of 1.0), but the median scores ranged between 0 and + 1.0 under congruent conditions. The agency statements were negatively rated through all incongruent conditions (medians were less than −0.5).

Because the Likert scale is ordinal, an aligned-rank transform was applied, and a three-way ANOVA was conducted considering the within-subject factors of hand avatar type (realistic human or non-human), congruency (congruent and incongruent vision-haptic stimuli), and delay (0, 50, 100, 200, or 400 ms). For the sense of body ownership, ANOVA revealed significant main effects from hand avatar type (*F*(1,26) = 16.27, *p* < 0.001, $\eta _{p}^{2}$ = 0.38) and congruency (*F*(1,26) = 10.60, *p* = 0.003, $\eta _{p}^{2}$ = 0.29). No significant differences were found in the other main effect or interactions. For the sense of agency, ANOVA revealed significant main effects from hand avatar type (*F*(1,26) = 7.90, *p* = 0.009, $\eta _{p}^{2}$ = 0.23) and congruency (*F*(1,26) = 17.06, *p* < 0.001, $\eta _{p}^{2}$ = 0.40). No significant differences were found in the other main effects or interactions.

## Discussion

In the present study, it was found that the existence of self-avatar motion modulates the perception of the near-threshold level of the force direction generated by an asymmetric oscillation. A force direction discrimination task was conducted where participants judged the direction of being twisted while holding a haptic device and seeing a self-avatar of the hand. The delays between the visual and somatosensory cues were varied by changing the self-avatar’s congruency of motion and appearance. The motion of the human hand avatar following the somatosensory stimulus at 50 ms decreased the correct judgement of force direction relative to that of the non-human object, while delaying 200 ms increased it. Signal detection analysis showed that these effects were due to reduced sensitivity and not merely from changes in response bias. Thus, to our knowledge, this is the first study showing that the specific delays between the visual and somatosensory cues influence the performance of the force discrimination task in the incongruent direction between the visuo-proprioceptive cues.

The questionnaire results demonstrated that the rating scores for body ownership were affected by both the appearance of the hand avatar and congruency. Previous studies have reported that the sense of body ownership is influenced by visuo-proprioceptive congruency^11^ and the appearance of a self-avatar^9,12^. Thus, the results of this study are in line with several studies stating that human virtual hands induced a greater sense of body ownership than various non-human objects^5,29^. In contrast, congruency and not the self-avatar affected rating scores for the sense of agency. Some previous studies have claimed that the sense of ownership and agency are dissociated^30^. There is some evidence that sense of agency originates in neural processes responsible for the motor aspects of action^31,32^. In this study, however, the task was not strongly involved with motor action. It is possible that the participants felt an illusory movement due to visuo-proprioceptive cues, especially the visual movement of the self-avatar. Thus, it was concluded that the sense of agency was influenced by congruency of motion rather than the appearance of the hand avatar.

While sensitivity was influenced by delays between the motion of the human hand avatar and somatosensory stimulus, the delays between the visual and somatosensory cues did not influence body ownership or agency in subjective ratings. Some studies have shown that ownership is induced even in the presence of asynchronous visuotactile stimulation when the fake body part is realistic and overlaps in space with its real body counterpart^33,34^. Furthermore, previous studies on visuo-haptic asynchrony have reported that haptic feedback is more sensitive to delay than visual feedback, and visual-haptic asynchronies from 20 to 50 ms were unnoticed^19,35–38^. Visual and tactile synchrony discrimination has been reported to not have a higher temporal resolution than other visuo-audio or audio-tactile discrimination. The temporal order judgment task between visual and tactile synchrony perception showed that the just-noticeable difference (JND) was 27 ms^39^. In addition, a visual cue should have appeared 32 ms earlier than it did to feel simultaneously between visual and tactile cues^36^. Thus, a delay of 0 ms in this study would not be felt simultaneously. Instead, it was speculated that a delay of 50 ms could be felt as if the visual movement of the self-avatar and twisted stimuli cooccurred. This could be attributed to the delay affecting only sensitivity, and not ownership or agency, in the subjective ratings.

In contrast, a delay of 200 ms showed the opposite tendency, but not a delay of 100 or 400 ms. A time window of less than 300 ms is critical for the multisensory integration processes constituting the self-body image^17^. A study on self-tickling sensation showed that the tickle rating increased significantly with increasing delay; a delay exceeding 200 ms induced lesser tickling than that of 100 ms^40^. Because 400 ms was outside the time window of multisensory integration, the participant may not feel that the self-avatar movement was caused by their hand and/or arm movement. In addition, it was speculated that a delay of 100 ms with no significant differences resulted from contamination of the effects of 50 ms and 200 ms.

Taken together with the result of the sensitivity and the subjective ratings, our study suggests that a delay of 50 ms influences the performance of the force discrimination in the incongruent condition of hand motion and force direction is independent of the subjective ratings of the sense of body ownership or agency. Our result shows that there were no differences in the subjective ratings of a sense of body ownership or agency among the delay conditions while the sensitivity was influenced by the combination of a delay of 50 ms in the incongruent condition with human hand avatar. Since the task for the participants was not to move the hand avatar, a sense of body ownership or agency may not be strongly induced; the median scores of the subjective ratings did not reach 2.0. This might be the reason for no difference in the subjective ratings. However, further studies are warranted to answer this question.

One might argue that since the hand-arm self-avatar disconnected from the torso leads to not a strong sense of body ownership or agency. However, the participants can learn the exact relationship between the avatar and stimulus movements on their right hand after a few seconds in the experiment. Because the connectivity of the hands with the torso does not influence the performance of object selection tasks^41^ and some off-the-shelf VR application adopted a hand avatar without forearm, disconnectivity is considered to be unrelated to sensitivity or subjective ratings.

The findings of our study must be interpreted within the frame of several limitations. First, the movements of the fingers were not tracked or reflected in the self-avatar, when the self-avatar hand was clasped to hold the device. Second, the condition where visual movement follows haptic stimuli was not tested. Third, the movement of the self-avatar in the experiment was apparent to participants. The visual direction was clearly discriminable when the perceptual threshold of force direction discrimination was applied. Lastly, the effect of prolonged exposure to visuo-haptic delays was ignored. The ratings of ownership and agency significantly improved with the prolonged delay between moving and seeing the visual consequences of that movement^42^.

## Methods

### Participants

For this study, 27 healthy paid volunteers were recruited, including 15 women and 12 men, who were all right-handed, with a mean age of [M] = 22.7 years and an age standard deviation of [SD] = 3.0 years. The sample size was chosen to be twice that of previous studies that also used asymmetric vibrations^43–46^. The recruitment of the participants and experimental procedures were approved by the ethics committee of the University of Tokyo (approval number: UT-IST-RE-191108-1) and were conducted per the principles of the Declaration of Helsinki. All the participants had a normal or corrected-to-normal vision, and none of them had previously reported tactile, neurological, or motor abnormalities. All participants provided written informed consent and were naive to the aim of the study.

### Apparatus

**Figure 3:**
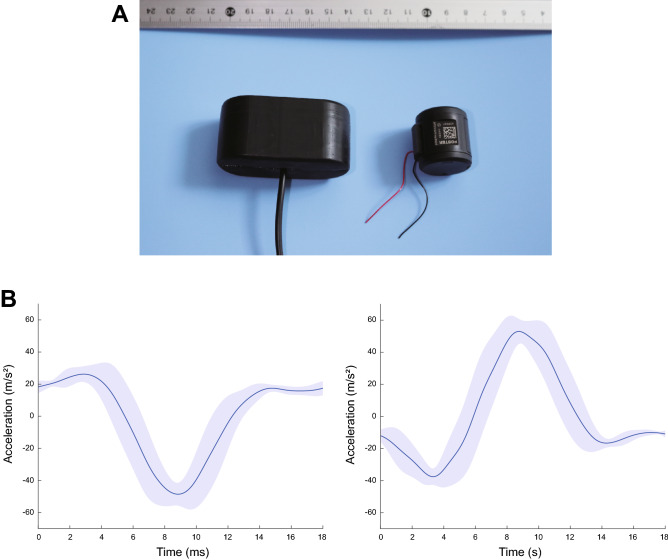
(**A**) Haptic device used in the experiment (left) and voice-coil actuator inside it (right). (**B**) Variation of the vibrator acceleration over one cycle producing either a forward (left) or backward (right) force sensation.

The experimental apparatus included an HMD (Vive Cosmos Elite, HTC), a position tracker (Vive Tracker 2018, HTC), a Windows-based computer (Intel i7-8750H, 16 GB RAM, and NVIDIA GTX1060), and a custom-made haptic device (Fig. 3A). The tracker was attached to a wristband on the participants’ right wrist. The position and orientation of the HMD and tracker were recorded using HTC’s Lighthouse system. The experimental program was developed using the Unity 3D platform (2019.4.15f) and ran at an average frame rate of 100 Hz.

The haptic device consisted of two voice-coil actuators (639897; Foster Electric Company), of the type used in PS5 DualSence controllers (Sony Interactive Entertainment Inc.), cased in a custom-made 61 mm × 32 mm × 29 mm box made of ABS resin. The gross weight of the haptic device was 50 g.

The delay time between the visual and haptic stimuli was measured in advance with a photodiode (Centronic BPW21) attached to the lens of the HMD for the visual stimuli and with an accelerometer (Analog Devices ADXL210) attached to the oscillator for the tactile stimuli. The timing of each stimulus presentation was adjusted to be within one frame (approximately 10 ms).

### Haptic stimuli

Several devices, such as gyroscopes^47–49^ and momentum wheels^50,51^, can create a twisting sensation. However, owing to the nonlinearity of human perception, a weak force stimulus is not clearly perceived, even when presented for a long period^43,52^. Considering the above, in this study, an asymmetric oscillation that induces an illusory force sensation and which can be easily achieved using off-the-shelf voice-coil type vibrators was selected as the source for the haptic stimulus. In such a method, vibrations with asymmetric acceleration and comprising intense pulses with longer periods of low-amplitude recovery, induce the sensation of being pulled or pushed in a particular direction despite the time-integrated forces in each direction being approximately equal^43,52–54^. Thus, by combining two or more vibrators oriented in different directions, their asymmetric oscillation can result in an illusory torque that creates the sensation of being twisted^43,45,55^.

In the case of the haptic device used in this research (Fig. 3A), two side-by-side vibrators generated asymmetric vibrations with synchronized peaks in opposite directions, so that when the left vibrator induced a forward force sensation, the right created a backward force feeling, leading the person holding the device to feel the sensation of being twisted clockwise; and vice versa. The asymmetric input signal was generated using a microprocessor (PIC18F252; Microchip Tech. Inc., Arizona, USA) in an additional custom-built controller connected to a computer via a USB port. Each signal was amplified using a power amplifier integrated circuit (HT82V739) with a maximum output voltage of 5 V. Finally, the amplified signal was input to the voice-coil actuators in the haptic device.

Figure 3B shows the acceleration caused by the vibrator over one 18-ms-long cycle. In the plot, the solid lines correspond to the average of 10 time-series measured for all the participants and smoothed using an infinite impulse response filter, while the shaded areas indicate the standard deviation.

It should be finally pointed out that, although active movement of the hand induces clearer illusions^56^, the asymmetric oscillation produces an illusory sensation of force without the need for actual movements of the user’s hand.

### Appearance of hand and forearm

**Figure 4:**
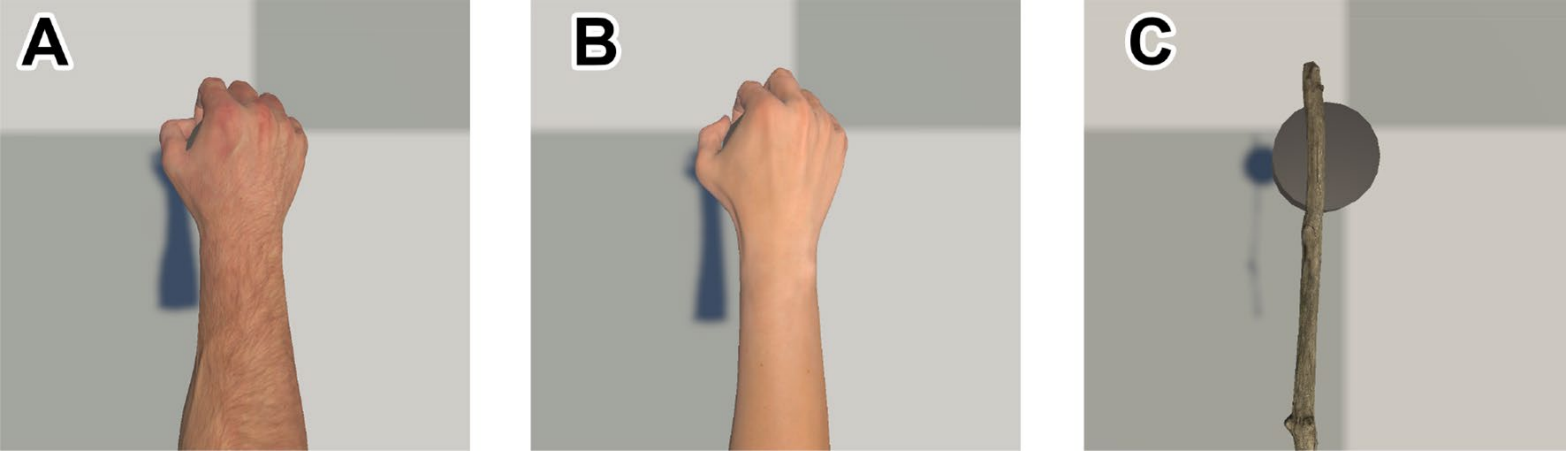
Two types of avatars were used in the experiment. For realistic hand avatars, gender-matching avatars (**A**: male, **B**: female) were implemented. For branch avatars (**C**), the length was identical to that of the realistic hand counterpart. Each avatar held a black object that imitated a haptic device.

Since the similarity between the appearance of self-avatars and that of the human body influences the sense of embodiment^8,9^, two types of avatar appearances were used: a realistic human hand and a branch, as shown in Fig. 4. In addition, the existence of the forearm plays an important role in judging direction because it reflects the rotational change more clearly than the hand alone. Hence, a realistic virtual hand and forearm were used for the realistic hand condition. The 3D model of the realistic hand was generated with the Leap Motion Software Development Kit, using male and female gender-matching (Fig. 4A,B), whereas the branch object was obtained from the Unity Asset Store (Fig. 4C). The length and end position of the avatars corresponded to the width and position of the palm of a realistic hand model. Both avatars held a model of the vibrator.

### Procedure

**Figure 5:**
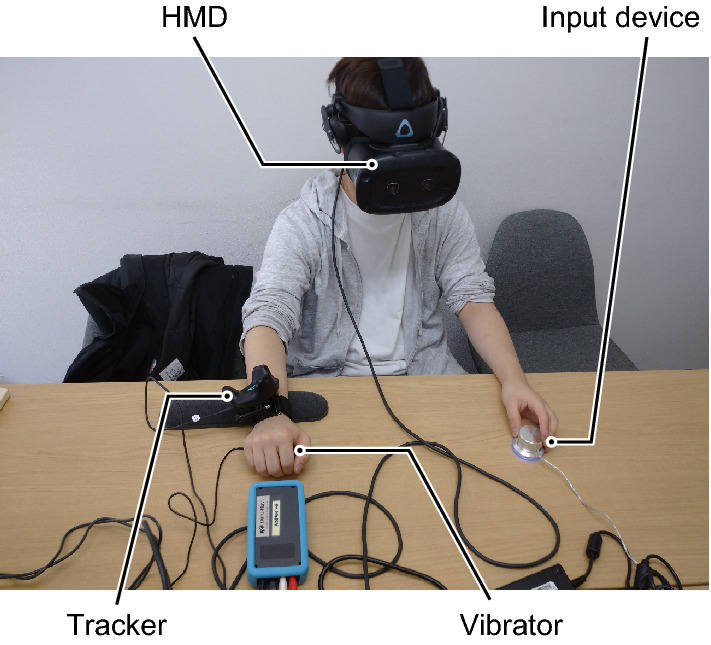
Experimental setup. The participants held the vibrator with their right hand and the control knob with their left hand. Hand movements were traced using the tracker on the right-hand wrist, which reposed on a rest cushion.

Participants were seated and made to wear an HMD and a wristband. They were asked to hold the haptic box in their right hand with the palm facing down, as shown in Fig. 5. The vibration amplitude threshold for direction discrimination in each participant was determined through a practice session prior to the main experiment. If participants did not give correct responses in between 70 and 80% of the practice trials, the amplitude was modified, and the process was repeated until a reliable threshold was found. Then, the participants performed practice trials to get acquainted with the task.

**Figure 6:**
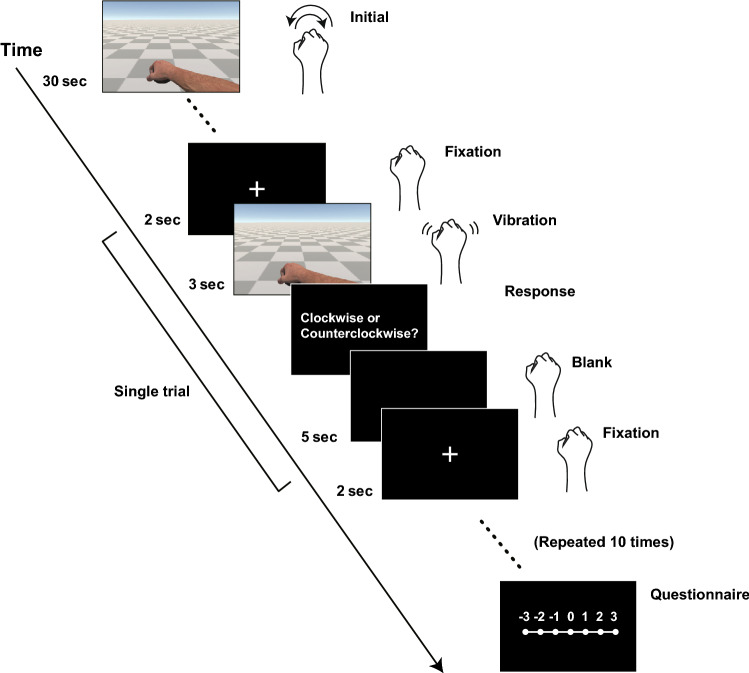
Timeline of a single trial during which visual and haptic stimuli are presented.

The main experiment included 22 blocks, each consisting of 10 trials. At the beginning of each block, the participants were asked to gaze at their avatar forearms and freely rotate their hands in the azimuth plane for 30 s. At the beginning of each trial, participants were instructed to fixate on a cross displayed on the HMD screen for 2 s. Then, the fixation cross disappeared, and visual and bodily stimuli were presented for 3 s. Next, the participants were asked to select the perceived twisting direction using a control knob (PowerMate, Griffin Technologies Inc.) placed on the left-hand side of the table. Lastly, after a 5 s blank, a fixation point appeared for 2 s, following which the participants were instructed to observe the hand avatar, before starting a new trial. Fig. 6 shows the timeline of a single trial.

A questionnaire was presented to the participants after every block (i.e., every ten trials), asking them to grade their level of agreement with the statements in the questionnaire. No feedback was given to the participants during the experiment.

During the “vibration” step of each trial, visual movements of the hand and forearm avatar, which consisted of $30^{\circ }$ rotations for 1 s, were congruent or incongruent with the twisting of the participant’s hand. The hand movement preceded the haptic stimuli, with onset differences of 0, 50, 100, 200, and 400 ms. A no-visual-movement cases were also included as a control condition.

Each participant performed 220 trials (two-hand avatar conditions × two congruent conditions × five delays × 10 trials; two-hand avatar conditions × haptic-only condition × 10 trials). The type of hand avatar, visual movement congruency, and delay were kept constant during all the trials in each of the 22 blocks. The order of the stimulus in the blocks was randomized for each participant. Participants were provided with at least a 3-min break after every block and could rest at any time to eliminate the influence of fatigue. The total experiment time for a typical participant was roughly 1.5 h.

### Questionnaire

The subjective feelings of body ownership and agency were measured at the end of each stimulus block using six items from established questionnaires^33,57^ supplemented by a Japanese translation. The included items, whose order was randomized for all the trials, are as follows:

Ownership

1. I felt as if the virtual hand (branch) was my hand.
2. It felt as if the virtual hand (branch) I saw was someone else’s.
3. I felt as if I had two right hands.

Agency

Q4. It felt I could control the virtual hand (branch) as if it was my own hand.
Q5. The movements of the virtual hand (branch) were caused by my movements.
Q6. I felt as if the virtual hand (branch) was moving by itself.

The participants responded to these items using a 7-point Likert scale, where −3 denoted strong disagreement, + 3 denoted strong agreement, and 0 referred to “not answerable”, i.e., “neither agree nor disagree”. Statements Q2, Q3, and Q6 were reverse scale questions. The subjective feelings of ownership and agency were quantified by the mean of items Q1, Q2, and Q3, and that of items Q4, Q5, and Q6, respectively.

### Data analysis

The existence of visual motion might influence either perceptual sensitivity or post-perceptual response bias. Signal detection theory offers a framework for distinguishing between sensitivity (d’) and bias (criterion) effects^58^. It was hypothesized that the simultaneous existence of a visual avatar motion and a haptic stimulus would yield a change in perceptual sensitivity. Regarding response bias, no specific predictions were made. Since for the application of signal detection theory, a signal should be predefined, the ‘counter clockwise’ direction was arbitrarily defined as the signal to be detected.

The subscales of ownership and agency ratings in the questionnaire were aggregated and averaged, inverting the answers for the reverse scale statements, to compute the score for each experimental block.

The Shapiro–Wilk normality test was initially conducted to determine whether the data followed a normal distribution. If the assumption of normality was violated, an aligned rank transform was applied to the sensitivity values to analyze the effects of interaction with nonparametric data^59^, and used $\eta _{p}^{2}$ to quantitatively compare the strength of such effects. Post hoc pairwise comparisons within the ART paradigm^60^ were made, applying the Holm correction to adjust p with a significance level of 0.05. All statistical tests were performed using the computing software MATLAB R2020a (The MathWorks, Massachusetts, USA) and R (v.4.1.2).

### Ethical statement

The experimental methods were approved by the Ethical Committee of the University of Tokyo, and performed in strict conformance with relevant guidelines and regulations.

## Supplementary Information

Supplementary Information.

## Data Availability

All data generated and analyzed during this study are included in this published article and its supplementary information files.
